# Age-Related Engagement and Outcomes in a Cancer Survivor Self-Management eHealth Program

**DOI:** 10.1093/geroni/igaa057.2240

**Published:** 2020-12-16

**Authors:** Corinne Leach, Shawna Hudson, Michael Diefenbach, Sicha Chantaprasopsuk, Catherine Alfano

**Affiliations:** 1 American Cancer Society, Atlanta, Georgia, United States; 2 Rutgers Robert Wood Johnson Medical School, New Brunswick, New Jersey, United States; 3 Northwell Health, Manhassett, New York, United States

## Abstract

While eHealth programs equip survivors with tools at where and when they need, their benefit to and engagement patterns among older adults are less known. Data come from the Springboard Beyond Cancer RCT, a cancer survivor self-management program (N=176; 88 control, 88 intervention arm) and the corresponding qualitative evaluation/user testing (N=40). Younger survivors, but not older, preferred socially interactive and personalized long-in features which enable greater tailoring of the program. However, the older survivors who did enroll in the RCT were equally as likely as their younger counterparts to engage with one or more aspects of program. Health self-efficacy improvement from baseline to 3 months was significant among younger participants in the intervention (p<.05) but not the control arm (p=.54) (d=.20) and marginally significant among older survivors (age 60+) in intervention (p=.06) but not the control arm (p=.58) (d=.28). Results suggest that the program may benefit survivors regardless of age. Part of a symposium sponsored by the Cancer and Aging Interest Group.

